# Changes in Aphid—Plant Interactions under Increased Temperature

**DOI:** 10.3390/biology10060480

**Published:** 2021-05-28

**Authors:** Jan Dampc, Mateusz Mołoń, Tomasz Durak, Roma Durak

**Affiliations:** 1 Department of Experimental Biology and Chemistry, University of Rzeszów, Pigonia 1, 35-310 Rzeszów, Poland; 2 Department of Biochemistry and Cell Biology, University of Rzeszów, Zelwerowicza 4, 35-601 Rzeszow, Poland; mateuszmolon@univ.rzeszow.pl; 3 Department of Plant Physiology and Ecology, University of Rzeszów, Rejtana 16c, 35-959 Rzeszów, Poland; tdurak@univ.rzeszow.pl

**Keywords:** insect physiology, plant physiology, enzymatic markers, environmental stress, climatic changes

## Abstract

**Simple Summary:**

The changing climate, particularly the temperature increase, can affect both herbivorous insects and plants. Aphids, being poikilothermal organisms, are directly exposed to an increase in temperature that, in turn, affects their biology. An increase in temperature can also indirectly affect aphids by changing the quality of the host plant tissues. This work focused on investigating the effects of climate change on plant–insect interaction. In particular, it was analyzed how an increase in ambient temperature can affect the condition of *Macrosiphum rosae* and its host *Rosa rugosa*, and how it correlates with the activity of oxidative stress-related enzymes (SOD, CAT, POD, β-glucosidase, GST, and PPO) both in insect and plant tissues. Thermal stress ranging from 25 to 28 °C has a significant impact on *M. rosae*–*R. rugosa* interaction, affecting aphid fitness and the activity of enzymes related to oxidative stress in both insect and plant.

**Abstract:**

Thermal stress in living organisms causes an imbalance between the processes of creating and neutralizing reactive oxygen species (ROS). The work aims to explain changes in the aphid–host plant interaction due to an increase in temperature. Tests were carried out at three constant temperatures (20, 25, or 28 °C). Firstly, changes in development of *Macrosiphum rosae* were determined. Secondly, the activity of enzymatic markers (superoxide dismutase (SOD), catalase (CAT), glutathione *S*-transferase (GST), β-glucosidase, polyphenol oxidase (PPO), and peroxidase (POD)) in aphid *M. rosae* tissues and host plant were analyzed at all temperatures. An increase in temperature to 28 °C had a negative effect on the biology of *M. rosae* by shortening the period of reproduction and longevity, thus reducing the demographic parameters and fecundity. Two stages of the aphid’s defensive response to short-term (24–96 h) and long-term (2 weeks) thermal stress were observed. Aphid defense responses varied considerably with temperature and were highest at 28 °C. In turn, for the plants, which were exposed to both abiotic stress caused by elevated temperature and biotic stress caused by aphid feeding, their enzymatic defense was more effective at 20 °C, when enzyme activities at their highest were observed.

## 1. Introduction

It has been predicted that the occurring climatic changes, including temperature increase, will affect the interactions between phytophagous organisms and host plant systems [1,2]. Aphids are poikilothermic organisms; therefore, temperature is the main factor influencing their development [1]. These insects can react to an increase in temperature by faster development [3,4,5], increased reproduction [1], higher winter survival [6,7], changes in life cycles [8], changes in migration timing [2,9], or changes in the dynamics of populations [10,11,12]. Aphids are an excellent model for studying the impact of climate change on insects, due to their short generation time, high reproductive rate, and telescopic development [2].

Increasing ambient temperature influences the developmental phases of aphids, as well as acts at cellular and metabolic levels. At a cellular level, it can disrupt mitochondrial function by affecting oxidative phosphorylation and cellular respiration. Thermal stress may disrupt the generation and scavenging of ROS (reactive oxygen species), causing oxidative stress (OS) [13,14]. In addition to endogenous sources, ROS may come from exogenous sources. These particles can be produced by plants to defend themselves against phytophagous organisms due to damage to plant tissues during aphid feeding [15,16]. Temperature rise has a direct effect on aphids, while it also affects the host plants that the aphids feed on [17]. Thermal stress in plants can affect photosynthesis efficiency, growth, pigmentation level, water loss, wilting, necrosis, and overgeneration of ROS [18,19].

The balance between ROS production and elimination at an intracellular level is strictly regulated, for example, by enzymatic mechanisms [20]. The first line of defense of aerobic organisms against OS involves antioxidant enzymes such as superoxide dismutase (SOD) and catalase (CAT), which are found in both aphids [15,21] and plants [22]. Detoxification enzymes such as β-glucosidase and glutathione *S*-transferase (GST) are multifunctional enzymes. The main function of these enzymes in aphids is the metabolism of xenobiotics to less toxic compounds [23,24]. In plants, these enzymes play an important role in defense. GSTs are enzymes involved in plant development, endogenous metabolism, detoxification of xenobiotics, and stress tolerance [25]. β-Glucosidases are involved in the release of toxic aglycans when plant tissues are damaged by feeding insects [26]. Oxidoreductive enzymes such as peroxidase (POD) and polyphenol oxidase (PPO) in aphids are responsible for neutralizing a wide spectrum of plant phenolic compounds [27]. In plants, however, they can catalyze the oxidation of phenolic compounds to quinones as a result of insect feeding and sweep away ROS. These enzymes also take part in lignification processes [28].

The rose aphid *Macrosiphum rosae* (Linnaeus, 1758) (Hemiptera: Aphidoidea) is a heteroecious, holocyclic species. It is an oligophagous insect that feeds on plants of the Rosaceae family [29]. It is often present in mass, causing significant damage such as deformation of shoots and leaves, as well as early defoliation and disturbance of inflorescence development. It is a serious pest for cultivated and ornamental plants [4,30]. The effect of temperature on the biological parameters of the rose aphid, a prevalent pest distributed worldwide, has been studied, taking into account e.g., various host plants [4,31,32].

The plant defense response can be induced by both biotic factors such as phloem-feeding insects and abiotic factors [33]. Our previous research showed the first information about the effect of temperature rise on complex plant–insect interactions, but little is known about the defense mechanisms of aphids [34]. In order to describe the defense mechanisms or response patterns of aphids, we performed experiments based on *M. rosae.* The presented work shows how an increase in ambient temperature affects aphids and the interaction between phloem-feeding insects and the host plant, as well as the response patterns of aphids. The following hypotheses were verified: (1) the increase in temperature triggers the two successive enzymatic defense stages of aphids; (2) an increase in temperature can shorten longevity and can limit fecundity and population growth; (3) The enzymatic defense of plants, infected by phloem feeding-insects, is more effective at lower temperatures.

## 2. Materials and Methods

### 2.1. Aphids

*Macrosiphum rosae* were obtained from the field and placed in an MLR-351H climate chamber (MLR-351H; Sanyo Corp., Osaka, Japan) under controlled conditions. Aphids for the experiments were propagated in a 16 h light/8 h dark photoperiod at 20 ± 1 °C, 60% ± 5% humidity. Only spring generation, wingless parthenogenetic females were used.

### 2.2. Host Plants

Two year old seedlings of *Rosa rugosa* were used as host plants. Plants were free of pathogens and planted in pots of 30 cm × 30 cm × 30 cm. Before starting the experiment, the plants were kept at 20 °C to acclimatize for 2 weeks. 

### 2.3. Entomological Experiments

All entomological experiments to check the effect of temperature on survival, fecundity, and development rate of insects fed on the host plant were carried out in a climate chamber (as above) under controlled thermal conditions at three temperatures: 20, 25, or 28 °C ± 1 °C at 60% ± 5% humidity in a 16 h light/8 h dark photoperiod. For most species of aphids, the optimal temperature for development is in the range of 20–25 °C, while a temperature close to 30 °C is lethal. We chose temperatures above the optimal *M. rosae* temperature to show potential changes in development as a result of global warming. We selected a temperature range that could be considered typical in a temperate climate.

#### 2.3.1. Longevity and Total Fecundity

The effect of thermal condition on longevity and the length of three phases (pre-reproduction, reproduction, post-reproduction of *M. rosae*) was measured. Twenty adult aphids were placed on a sprig of the host, and their offspring were included in the experiment. The first 25 nymphs born were taken and protected individually with a fine mesh isolator and monitored to maturity. When the females reached maturity and began to give birth, all newborn nymphs were counted once a day with a soft brush and removed. The experiment ended when the last adult aphid died. On the basis of the observations made, the length of pre-reproduction, reproduction, and post-production, total fecundity, and longevity were calculated.

#### 2.3.2. Demographic Parameters, Survival, and Average Daily Fecundity

The experiment was conducted at three constant temperatures (20, 25, or 28 °C). Five plants of *R. rugosa* were placed under controlled conditions in climate chambers. Five adult *M. rosae* were placed on each plant until the birth of the nymphs. The adult aphids were removed, and 20 newborn nymphs (*n* = 100) were left on each plant. A fabric isolator was applied to the plants with insects. The development of all individuals was monitored until their death. Newborn nymphs were counted and removed daily. During the experiment, we collected data and calculated the survival, average daily fecundity of females, and demographic parameters of the population according to the following equations [35,36]:

- intrinsic rate of increase, rm = (lnMd × 0.738)/D,
  where D is the developmental period from birth to the beginning of the first reproduction (pre-reproductive period), and Md is the number of nymphs produced by the adult in the first D days of reproduction after the adult molt;
- net reproduction rates, Ro = Ʃ(lxmx),
  where lx and mx are cumulative daily survival and fecundity, respectively;
- finite rate of increase, λ = erm, where e is the base of the natural logarithm;
- mean generation time, T = ln Ro/rm;
- population doubling time, DT = ln 2/rm.

### 2.4. Biochemical Analyses 

#### Effect of Temperature on the Enzymatic Activity in Aphid and Plant Tissues

The experiment was carried out independently at three temperatures (20, 25, or 28 °C) under constant conditions as previously described. Thirty adult aphids *M. rosae* were placed on each plant. Aphids fed on the host plant for 24, 48, 72, 96, or 336 h (2 weeks). A control (0 h) sample was collected before starting each experiment. The enzyme activity was determined in plants infected with aphids and, in parallel, in plants not infected with aphids, grown as an independent control, at each temperature. After the experimental time, aphids (30 aphids in a sample) and leaves on which they were fed (1 g of plant material) were collected and frozen in liquid nitrogen. The experiment was carried out in three replications. Samples were kept at −85 °C (Deep freezer VXS 490, Thermo Scientific, Berlin, Germany) until analysis. 

Frozen insects (30 individuals) were flooded with phosphate buffer (0.1 M, pH 7.0) and homogenized at 0 °C. The resulting homogenate was centrifuged (Eppendorf Cen-trifuge 5810 R) at 4 °C. Plant material (1 g) was homogenized in phosphate buffer (0.1 M, pH 7.0) at 0 °C. The homogenate was centrifuged at 4 °C. The collected supernatant was used for enzymatic analyses according to the procedure described by Dampc et al. [34].

The activity of superoxide dismutase (SOD) was measured using a standard method according to Wang et al. [37]. Catalase (CAT) activity was determined using the standard method described by Aebi [38]. β-Glucosidase activity was determined by the reaction described by Katagiri [39]. Glutathione *S*-transferase (GST) activity was measured according to Leszczyński and Dixon [40]. The level of polyphenol oxidase (PPO) was measured with the method described by Miles [41] with the Laurema et al. modification [42]. Peroxidase (POD) activity was determined using the method of Fehrman and Dimond [43]. The protein content in aphid and plant extracts was determined by a standard method based on the biuret reaction according to Lowry [44].

### 2.5. Statistical Analyses 

We tested normality using the Shapiro–Wilk and the homogeneity of variance using the Levene test. Data presented in the study are presented as means with standard error values (mean ± SE). The significance of differences between life-cycle length and fecundity at individual temperatures was tested by the Kruskal–Wallis test. All biochemical analyses were performed in three independent replicates (*n* = 3). Analysis of variance (ANOVA) was used to test differences among average enzymatic activity. To compare the averaged values of enzymatic activity, we used a two-way ANOVA test for aphid tissues and a three-way ANOVA test for plant tissues, with statistical significance at *p* < 0.05. Averaged values of enzymatic activity were compared using two explanatory variables (factors) for aphids temperature (20, 25 or 28 °C) and time (0, 24, 48, 72, 96 and 336 h), and three explanatory variables for plants, temperature, time and presence of aphids. The analyses were performed separately for each enzyme. The significance of differences among the average enzymatic activity indices was calculated with Duncan’s multiple range test. Statistical significance was estimated at *p* < 0.05. All statistical analyses were done using Statistica version 13 (TIBCO Software Inc., Palo Alto, CA, USA, 2017).

## 3. Results

### 3.1. Entomological Experiments 

**Figure 1:**
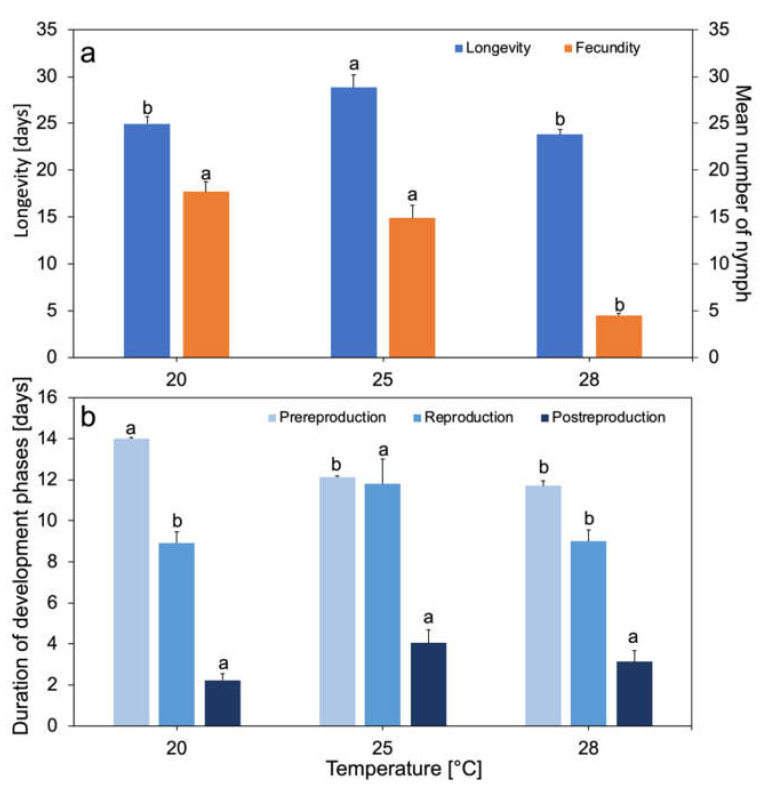
Developmental time and fecundity of apterous *Macrosiphum rosae* as a function of temperature. Longevity time and fecundity (*n* = 25 at each temperature 20, 25, and 28 °C) (**a**). Time of developmental phases of apterous *M. rosae* (pre-reproduction, reproduction, and post-reproduction) (**b**). Values marked with different letters differ significantly at *p* < 0.05, for each parameter (Kruskal–Wallis test).

The duration of the development phases and longevity of *M. rosae* changed with the increase in ambient temperature. The average total longevity of females of this species ranged from 23.8 to 28.8 days in the analyzed temperature range (Figure 1a). Aphids lived the longest at 25 °C, an intermediate duration at 20 °C, and the shortest at 28 °C. Significant differences in longevity were observed at 20 and 25 °C (*p* < 0.01) and 25 and 28 °C (*p* < 0.01), while no differences were found between 20 and 28 °C (Figure 1a). The pre-reproduction phase shortened from 14 days at 20 °C to 11.4 days at 28 °C. Significant differences in the length of this phase were shown between individuals at 20 °C and 25 °C (*p* < 0.001) and at 20 °C and 28 °C (*p* < 0.001) (Figure 1b). The average reproduction time at 20, 25, and 28 °C was 8.9, 13, and 9 days, respectively. The reproduction time of aphids living at 25 °C was the longest. Significant differences in the length of this phase between 20 and 25 °C (*p* < 0.01) and between 25 and 28 °C (*p* < 0.01) were noted (Figure 1b). The average post-reproduction time slightly increased with increasing temperature at 25 °C. It was 2 days at 20 °C, 4 days at 25 °C, and 3 days at 28 °C. There were no statistically significant differences in post-reproduction phase length between temperatures (Figure 1b). Aphids living at 20 and 25 °C gave birth to an average of 18.0 and 17.0 nymphs, respectively. Aphids living at 28 °C gave birth to an average of 4.5 nymphs (Figure 1a). Aphids gave birth to a maximum of 25 nymphs at 20 °C, 29 nymphs at 25 °C, and 10 nymphs at 28 °C, and a minimum of eight, five, and one nymph, respectively. Statistically significant differences in mean fecundity were demonstrated between individuals at 20 and 28 °C (*p* < 0.001) and at 25 and 28 °C (*p* < 0.001), while no significant differences were found in the fecundity of females living at 20 or 25 °C (Figure 1a).

**Figure 2:**
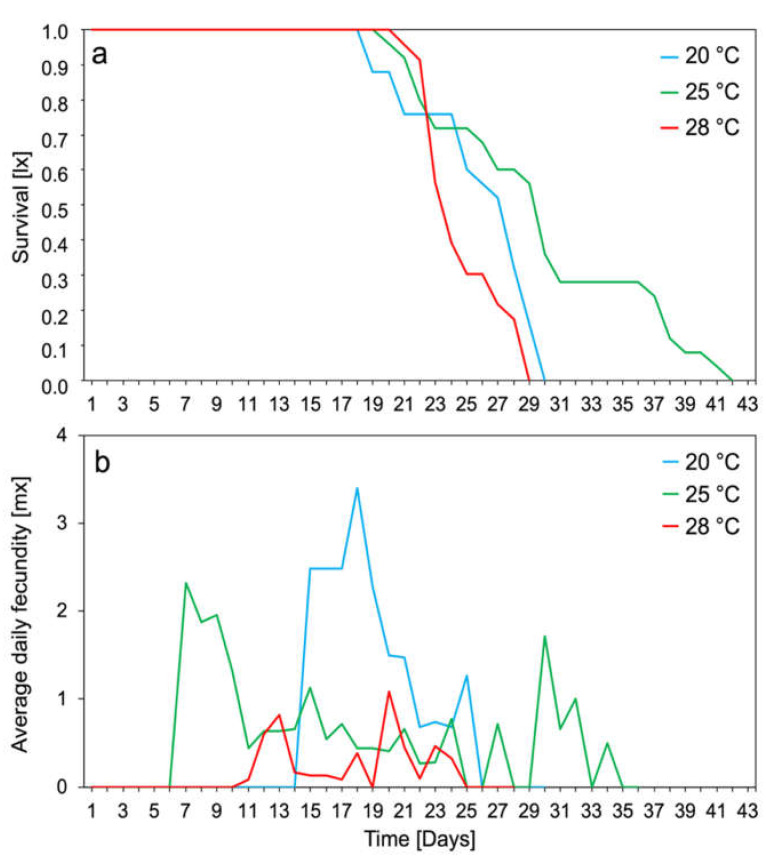
Survival rates (**a**) and daily fecundity (**b**) of apterous females *Macrosiphum rosae* at different temperatures (*n* = 100 at each temperature: 20, 25, and 28 °C).

The survival of the *M. rosae* population was strongly dependent on the temperature and was the highest at 25 °C (Figure 2a). At each temperature tested, all nymphs survived up to the reproduction period. Additionally, adults showed the highest average daily fecundity at 20 °C, which was about 3.5 nymphs per female (Figure 2b).

The calculated demographic parameters for population of *M. rosae* showed that the intrinsic rate of increase (rm) reached a minimum (0.09) at 28 °C and a maximum (0.16) at 20 and 25 °C. The finite rate of increase (λ) showed that the population of *M. rosae* increased 1.17-fold during the day at 20 and 25 °C, while they increased 1.09-fold at 28 °C. The generation time (T) was the shortest at 28 °C (15.62 days) and the longest at 20 °C (17.94 days). The net reproductive rate (Ro) decreased from 17.36 at 20 °C to 4.08 at 28 °C. Doubling time (DT) was the longest at 28 °C (7.7 days), while, at 20 and 25 °C, it was shorter and amounted to 4.33 days (Table 1).

### 3.2. Biochemical Analyses 

#### 3.2.1. Superoxide Dismutase (SOD) and Catalase (CAT) Activity in Aphid and Plant Tissue

**Figure 3:**
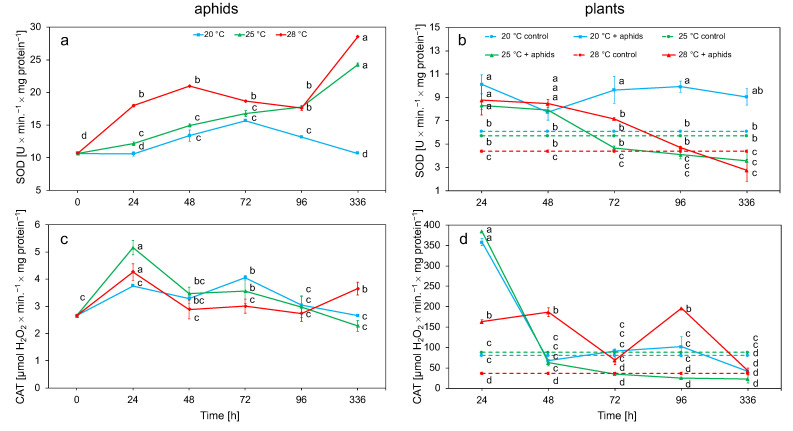
Changes in the SOD and CAT activity (means ± SE) in the tissues of aphid *Macrosiphum rosae* (**a**,**c**) and plant *Rosa rugosa* at different temperatures and during aphid feeding (**b**,**d**). Values marked with different letters differ significantly at *p* < 0.05 (Duncan test).

The analysis of SOD activity in aphid tissues showed a significant increase in activity depending on temperature; this enzyme showed the highest activity values at 28 °C and the lowest at 20 °C. SOD activity increased at 25 and 28 °C throughout the experiment. At 20 °C, after a slight increase in activity after 48 h, it decreased, while it was comparable with the control after 336 h. In turn, after 2 weeks, SOD activity at 25 and 28 °C reached the highest values (Figure 3a). CAT activity at 25 and 28 °C reached the highest values after 24 h and then decreased to the end of the experiment. At 20 °C, the activity of this enzyme gradually increased to 72 h and then decreased. After 2 weeks at all temperatures, the activity decreased only at 28 °C, where a slight increase in activity was observed (Figure 3c). The activity of antioxidant enzymes was dependent on temperature and time (Table 2).

Analysis of the enzymatic activity in plant tissue showed that the increase in temperature caused an increase in SOD activity in *R. rugosa* tissues infected by aphids from 24 h, in comparison to the control. An increase in activity was observed at all temperatures, with the highest value at 20 °C. Activity at any temperature gradually decreased during the time period of the experiment (Figure 3b). At 28 °C, the highest activity was maintained up to 48 h, while, at 20 °C, high activity was observed throughout the experiment. CAT activity in rose tissues reached its highest values after 24 h at 20 and 25 °C; these values after 48 h were reduced to a level comparable to the control at 20 and 25 °C. At 28 °C, the elevated CAT level, compared to the control, was maintained for the first 4 days. After 2 weeks, the activity showed a value comparable to that of the control (Figure 3d). SOD and CAT activity in plants infected with aphids was higher than in control plants. The activity of SOD and CAT in plant tissue was dependent on temperature, time, and aphid feeding (Table 2).

#### 3.2.2. Glutathione *S*-Transferase (GST) and β-Glucosidase Activity in Aphid and Plant Tissue

**Figure 4:**
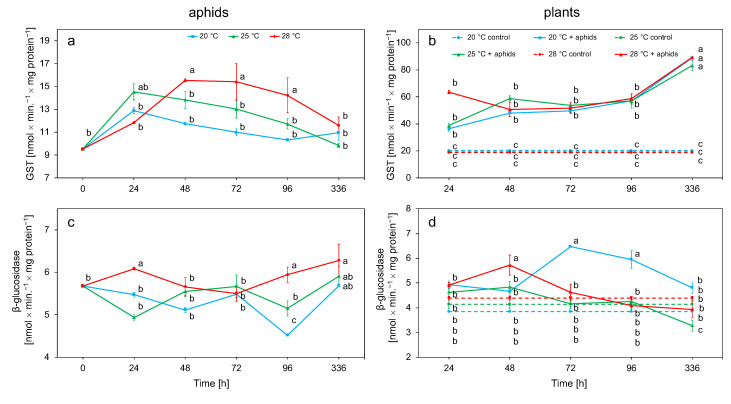
Changes in GST and β-glucosidase activity (means ± SE) in the tissues of *Macrosiphum rosae* (**a**,**c**) and *Rosa rugosa* at different temperatures and during aphid feeding (**b**,**d**). Values marked with different letters differ significantly at *p* < 0.05 (Duncan test).

A significant increase in GST activity in aphid tissues was observed at 25 °C after 24 h and at 28 °C at 48 h, after which a decrease was observed. The highest activities were observed at 28 °C (Figure 4a). β-Glucosidase showed an increase in activity after 24 h only at 28 °C, after which the initial increase activity decreased. This enzyme reached its highest value after 2 weeks at each temperature. The enzyme activity reached the lowest activity values at 20 °C (Figure 4c). Both temperature and exposure time influenced the activity of these enzymes (Table 2).

The activity of GST at all temperatures was significantly higher in plants infested with aphids than in control plants. Activity at each temperature remained stable throughout the experiment and reached the highest values 2 weeks after the experiment. There were no significant differences in GST activity between the temperatures on the individual days of the experiment in plants with aphids (Figure 4b). The activity of β-glucosidase increased for 48 h at 25 and 28 °C, while, at 20 °C, it increased for 72 h. At 20 °C, β-glucosidase activity was higher than in control plants for the first 4 days; thereafter, activity decreased to values comparable with the control in two weeks. After the initial increase at 25 and 28 °C in 48 h, the activity rapidly decreased to the control value (Figure 4d). Detoxification activity of plant enzymes was dependent on temperature, aphids, and time (Table 2).

#### 3.2.3. Polyphenol Oxidase (PPO) and Peroxidase (POD) Activity in Aphid and Plant Tissue

**Figure 5:**
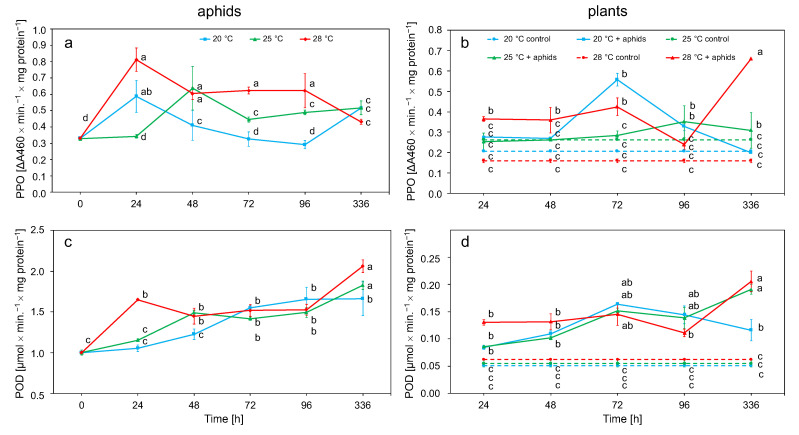
Changes in the PPO and POD activity (means ± SE) in the tissues of *Macrosiphum rosae* (**a**,**c**) and *Rosa rugosa* at different temperatures and during aphid feeding (**b**,**d**). Values marked with different letters differ significantly at *p* < 0.05 (Duncan test).

The PPO activity in insect tissues was directly proportional to the temperature increase, with the highest activity values obtained at 28 °C. This enzyme gradually decreased its activity after an initial increase after 24 h. Only at 25 °C did the activity increase slightly up to 72 h before decreasing (Figure 5a). POD activity increased at all temperatures to reach peak values after 2 weeks of the experiment. The highest activity values were observed at 28 °C and the lowest values were observed at 20 °C (Figure 5c). The activity of PPO and POD in aphids depended on the temperature and duration of exposure to the stress factor (Table 2).

PPO activity in plant tissue increased most rapidly at 20 °C, reaching the highest value at this temperature after 72 h. At other temperatures, the increase was slower. The increase in activity at 25 °C and 28 °C was less rapid, whereby the highest activity at these temperatures were achieved after 2 weeks (Figure 5b). The increase in POD activity was observed at all temperatures (20, 25, and 28 °C) and was maintained for the first 3 days with a slight decrease in values after 96 h. During this time, the greatest increase in POD activity was observed at 20 and 25 °C. After 2 weeks at 25 and 28 °C, another increase in activity was observed, while, at 20 °C, a slight decrease in activity was noted. Throughout the course of the experiment, the POD activity was higher than that of the control plants. (Figure 5d). The activity of these enzymes in plant tissues was influenced by temperature, time, and aphids (Table 2).

## 4. Discussion

Temperature increases can affect both insects and plants, inducing thermal stress. The effect of thermal stress on organisms is the disruption of the homeostasis of ROS production and scavenging, which can lead to oxidative damage [14]. Due to the coordinated action of antioxidant, detoxification, and redox enzymes, insects and plants neutralize ROS and harmful metabolites [45,46]. Plants, as a result of exposure to one stress, may modify the ability and intensity of the response to a subsequent stress factor by modifying the metabolic level [47].

Temperature for poikilothermic insects is one of the most important environmental factors influencing the rate of development, reproduction, and survival [48]. The studied Polish population of *M. rosae* had the longest longevity at 25 °C and the shortest at 28 °C, which was about 20% shorter (Figure 1a). The shortening of longevity with increasing temperature was also observed in populations originating from Turkey and Iran, with aphids originating in Turkey having a higher maximum longevity than those originating in Iran [4,42]. The Polish population of *M. rosae* showed the highest fecundity at 20 and 25 °C, respectively; the females gave birth to an average of 18 and 17 nymphs (Figure 1a). The Turkish and Iranian populations showed the highest fecundity at 22.5 °C of 35 and 29 nymphs, respectively [4,31]. Discrepancies in female longevity and fecundity were probably caused by the inter-population differences of populations developing in different climate zones, as seen in, for example, *Drosophila melanogaster* [49]. An increase in temperature up to 28 °C caused a threefold decrease in female fecundity (Figure 1a). Temperature increase also caused *M. rosae* to reduce its pre-reproduction phase; its reproductive phase was longer at 25 °C and shorter at 28 °C (Figure 1b). It was also observed that the increase in temperature decreased the survival and daily fecundity of aphids (Figure 2). The demographic parameters of the rose aphid population were the highest at temperatures of 20 and 25 °C, which was the optimum temperature for this population. However, an increase in temperature up to 28 °C lowered the value of demographic parameters of the population (Table 1). Temperature increase has a positive effect on the development of aphids, if it is within the tolerance limits of the species. For temperate climate species, the temperature of 28 °C is above the thermal optimum and has a negative effect [50,51,52,53]. An increase in temperature above the thermal optimum in *Myzus varians* disturbed its development in such a way that the aphids did not reach maturity and did not give birth to nymphs [54]. Additionally, high temperatures affect insects indirectly. The increase in temperature in the summer period adversely affected the obligate gut bacterium of *Nezara viridula*, which negatively affected their condition [55]. The elimination of endosymbionts such as *Buchnera*, observed in the aphids, was responsible for the low survival rate of aphid nymphs at high temperatures [56]. It has also been shown that, in the aphid–*Buchnera* mutualism, a point mutation in the *Buchnera* genome was related to the thermal tolerance of the host insect [57]. This may indicate that an increase in temperature up to thermal optimum not only causes disturbances in the development of aphids and lowers their longevity, fecundity, and demographic parameters, but also disturbs the mutualism between the aphids and their symbionts [54].

It is important to neutralize the negative effects of stress and the proper functioning of physiological and metabolic processes. Antioxidant, detoxification, and redox enzymes in aphids play a key role in defense. These enzymes are responsible for the neutralization of ROS and xenobiotics [46]. Temperature rise can also influence the physiological and metabolic changes of the host plant. Thermal stress can cause pigmentation loss, water loss, and photosynthesis disorders, as well as lead to overgeneration of ROS [18,19,22]. Plants, in order to eliminate oxidative damage to proteins, membranes, lipids, or nucleic acids, similarly to insects, create a coordinated system of antioxidant, detoxification, and redox enzymes that neutralize harmful metabolites and ROS [45].

Two stages of the aphid’s defense response to short-term (24–96 h) and long-term (2 weeks) thermal stress were observed. The first stage of defense against ROS involves cooperating antioxidant enzymes. SOD activity in *M. rosae* tissues was the lowest at 20 °C and, at this temperature, the activity of this enzyme increased the slowest and stabilized the fastest. In contrast, at 25 and 28 °C, activity increased over time and no decrease in activity was observed (Figure 3a, Table 2). An increase in CAT was observed after 24 h of the experiment. The activity of this enzyme gradually decreased over the course of the experiment; however, at 28 °C, it increased again after 2 weeks (Figure 3c, Table 2). SOD and CAT activity in *M. rosae* tissues increased in direct proportion to temperature. It was observed that an increase in temperature ranging from 20 to 28 °C resulted in an increase in aphid defense responses. Similar relationships occurred in *Aphis pomi,* due to the temperature increase. This oligophagous species showed a similar response to temperature rise over the same range as indicated by the similar SOD and CAT activity in this species compared to *M. rosae* [34]. An increase in the activity of SOD and CAT was also observed in *Sitobion avenae* and *Rhopalosiphum padi* due to the change of the host plant; after feeding on resistant cultivars, the level of these enzymes in the tissues of these species increased [16]. A change in SOD and CAT activity was observed as a result of the adaptation of the highly polyphagous *Myzus persicae* to different host plants [58]. An increase in temperature above the optimum in an insect *Mythimna separata* with chewing moths also resulted in an increase in the activity of SOD and CAT [59].

SOD and CAT play an important role in plant resistance to phytophagous insects and pathogens [60,61]. The increase in temperature and aphid feeding caused an increase in SOD activity in the first day. The plant’s defense reactions were most effective at 20 °C (Figure 3b, Table 2). Similarly, it was observed that the effectiveness of the plant’s defense reactions was highest at 20 °C in *Chaenomeles japonica* on which *A. pomi* was fed [51]. The relationship between SOD activity and the level of O_2_^•−^ due to feeding of *Diuraphis noxia* was observed in barley and wheat seedlings [62] and after feeding with *Cinara tujafilina* in *Thuja orientalis* tissues [63]. The increase in CAT activity in *R. rugosa* tissues infested by aphids was most visible after the first day of the experiment and had the highest values at 20 and 25 °C, which suggests that this enzyme works more effectively at lower temperatures. In control plants, slight differences in CAT activity were observed between 20 and 25 °C, and, at 28 °C, the activity was slightly lower. The small difference in temperature probably had little effect on the ROS level (Figure 3d, Table 2). Studies on *Glycine max* have shown that a temperature increase of about 2–3 °C has little effect on the plant [64]. A decrease in CAT activity in some plants has also been demonstrated as a result of high temperature [65]. A low CAT level allows the plant to maintain a higher concentration of H_2_O_2_, which adversely affects aphid feeding [60,66,67]. Research on resistant wheat cultivars showed a higher level of CAT than in cultivars susceptible to heat stress [68]. Plant cultivars resistant to phytophagous showed higher CAT activity than sensitive cultivars [69,70].

In aphids, detoxification enzymes (GST and β-glucosidase) play the main role in preventing the impact of changes in the host plant and neutralization of secondary metabolites [71]. Secondary plant metabolites for insects have toxic or allelopathic properties [72]. GST and β-glucosidase activity increased with increasing temperature in *M. rosae*; however, a significant increase in activity was only observed at 28 °C (Figure 4a, Table 2). The increase in GST activity was probably due to the accumulation of lipid peroxidation products due to the temperature increase at 28 °C [14,73]. The increase in GST activity is an indicator of aphids’ adaptation to changes in the composition of xenobiotics containing phenolic compounds of plant origin [27]. An increase in GST activity was observed as a result of temperature increase in *Aphidius gifuensis* [74]. The β-glucosidase activity is strongly related to the chemical composition of the host plant. Changes in the activity of GST and β-glucosidase were observed as a result of changing the host from primary to secondary in *R. padi* [75]. Similar relationships were observed in *C. tujafilina* after changing the host plant [71]. The increase in β-glucosidase activity, especially evident at 28 °C in *M. rosae* tissues (Figure 4c, Table 2), was probably related to changes in the sugar metabolism in the plant and, thus, the carbohydrate composition in the ingested food, which was evident after 96 h feeding [34]. Ambient temperature can influence the sugar metabolism in the plant, whereby an increase in temperature can cause starch breakdown [18,19]. Similarly, the increase in temperature caused an increase in β-glucosidase activity in the tissues of *Eurygaster maura* [76].

In the plant, GST activity gradually increased over time, probably due to the slow accumulation of lipid peroxidation products. The activity of this enzyme in plant tissue was high and similar at all temperatures during experiments, but we observed a significant increase after 2 weeks (Figure 4b, Table 2). Throughout the course of the experiment, the activity of GST in plants infested with aphids was higher than in control plants. *Rhopalosiphum padi* aphid feeding influenced the overexpression of GST isoforms in *Zea mays* seedlings [77], and an increase in GST expression was observed due to *M. persicae* feeding in *Arabidopsis thaliana* substances [78]. One of the functions of plant β-glucosidases is the hydrolysis of the β-glucosidic bond, thereby activating glycosides and releasing plant defense substances [79]. For the activation of glycosidic defense compounds, it is necessary to mechanically damage the cells so that the β-glucosidases can be activated. During penetration, aphids caused little damage to plant cells [80], leading to a slight increase in the activity of this enzyme. The highest activity was observed after 48 and 72 h at 20 °C, i.e., after longer feeding of insects (Figure 4d, Table 2). Increased β-glucosidase activity was observed in *T. orientalis* tissues after feeding with *C. tujafilina* [63] and *Ch. japonica* after *A. pomi* feeding [51]. 

The main task of redox enzymes in aphids is the conversion of toxic plant phenolic secondary metabolites into less toxic compounds, which are absorbed by insects with their food [81]. Plants in the first stage of defense against insects generate H_2_O_2_ and accumulate phenolic compounds, and then antioxidant and detoxification mechanisms are activated to neutralize the harmful effects of these substances on themselves [67]. The activity of PPO and POD in *M. rosae* tissues increased with increasing temperature. POD activity increased throughout the course of the experiment (Figure 5a,c, Table 2). Long-term thermal stress may lead to disturbances in the metabolism of phenolic compounds in plants, as indicated by the gradual increase in PPO and POD in rose and *M. rosae* tissues, as well as similar relationships observed in *A. pomi* [34]. Changes in phenol metabolism can lead to an increased generation of quinones, which are more toxic to aphids [82]. An increase in the activity of these enzymes was observed by feeding a diet containing geramine to aphids [83]. The observed change in PPO and POD activity was due to the change of the host plant [71].

It was observed up to about 72 h of the experiment that the increase in activity of POD and PPO was highest in plants at the temperature of 20 °C. The level of activity then decreased and increased again after 2 weeks. The greatest increase in activity during this time was observed at 28 °C in both POD and PPO (Figure 5b,d, Table 2). In addition to the ROS detoxification function, POD is also responsible for the formation of suberins involved in the repair of mechanical damage to the plant and involved in the formation of dehydrodiferous bridges in the cell wall [84]. PPO reduces the nutritional value of plants by oxidizing phenolic compounds to quinones, which, due to crosslinking with nucleophilic chains of proteins and free amino acids, makes plants less digestible for insects [85]. POD activity correlates with the feeding time and population size of *Acyrthosiphon pisum* [86]. Mechanical injuries during aphid feeding increase the POD activity [87]. Plants overexpressing POD-encoding genes showed increased mortality from larvae of *Helicoverpa zea* and *Spodoptera frugiperda* that fed on them [88]. These enzymes may adversely affect the number of feeding insects; as a result of their action, phenolic oxidation products dissolved in water may enter the gastrointestinal tract of insects, which may lead to the generation of ROS in the insects [89,90].

Long-term exposure to high temperatures (28 °C) affects the physiological response of aphids, thus limiting population development and growth. An increase in temperature above the optimum has a negative effect on the life cycle and the voltinism of insects [7,50]. Higher temperatures in some cases may be beneficial for aphids, which is especially visible in species from warmer climates and cosmopolitan species [2]. The first stage of the response of *M. rosae* to high temperature occurs in response to short-term exposure to heat (28 °C). Due to the coordinated action of antioxidant, detoxification, and redox enzymes, aphids prevent the formation of excessive ROS, which was also observed in *A. pomi* [51]. The second stage reaction is caused by the influence of the host plant subjected to long-term biotic and abiotic stress. Thermal stress and aphid feeding influenced the biochemistry of the host. Increased defense mechanisms of plants, which resulted in the increased defense of aphids, finally resulted in an increase in enzymatic activity [34]. Redox and detoxification enzymes play a major role in the second stage as they neutralize harmful plant xenobiotics. Plant defense responses varied with temperature and were highest at 20 °C, suggesting that, at this temperature, plants had the highest defense against heat damage and insects. A reduction in the plant’s defense response to stress can lead to an increase in damage caused by pests [51].

## 5. Conclusions

An increase in temperature to 28 °C had a negative effect on *M. rosae*, by shortening its period of reproduction and longevity, thus reducing demographic parameters and fecundity. The defense responses of aphids and plants differed significantly with temperature and were highest at 28 °C in aphids and at 20 °C in plants. The aphid defense responses occurred in two stages. The first stage was the response to short-term exposure to heat (28 °C), whereas the second was the aphid’s defense response to changes in the host plant subjected to long-term abiotic and biotic stress. Temperature is a key factor influencing plant–aphid interactions and physiological response, thereby possibly limiting the development of aphids.

## Figures and Tables

**Table 1:** Life parameters describing *Macrosiphum rosae* as a function of temperature.

Temperature	20 °C	25 °C	28 °C
Intrinsic rate of increase (rm)	0.16	0.16	0.09
Net reproductive rate (Ro)	17.63	14.76	4.08
Finite rate of increase (λ)	1.17	1.17	1.09
Generation time (T)	17.94	16.82	15.62
Doubling time (DT)	4.33	4.33	7.7

**Table 2:** Analysis of enzymatic activity in the tissues of aphid *Macrosiphum rosae* and plant *Rosa rugosa*. ANOVA was used to test differences between average enzymatic activity in different conditions (*p* < 0.05). (T, temperature; t, time; a, aphids).

	SOD	CAT	GST	β-Glucosidase	PPO	POD
**Aphid Tissue**						
T	F_(2,36)_ = 461.22 ***	F_(2,36)_ = 1.68 *	F_(2,36)_ = 2.86 *	F_(2,36)_ = 4.74 **	F_(2,36)_ = 5.18 **	F_(2,36)_ = 5.23 **
t	F_(5,36)_ = 240.31 ***	F_(5,36)_ = 12.26 ***	F_(5,36)_ = 3.14 *	F_(5,36)_ = 2.25	F_(5,36)_ = 3.37 **	F_(5,36)_ = 18.43 ***
T × t	F_(10,36)_ = 75.69 ***	F_(10,36)_ = 2.42 *	F_(10,36)_ = 0.79	F_(10,36)_ = 1.07	F_(10,36)_ = 2.40*	F_(10,36)_ = 2.27 *
**Plant Tissue**						
T	F_(2,60)_ = 26.28 ***	F_(2,60)_ = 12.21 ***	F_(2,60)_ = 0.58 **	F_(2,60)_ = 2.40	F_(2,60)_ = 0.85	F_(2,60)_ = 2.83
t	F_(4,60)_ = 5.60 ***	F_(4,60)_ = 181.09 ***	F_(4,60)_ = 7.98 ***	F_(4,60)_ = 1.16	F_(4,60)_ = 2.21	F_(4,60)_ = 5.70 ***
a	F_(1,60)_ = 21.02 ***	F_(1,60)_ = 238.87 ***	F_(1,60)_ = 467.29 ***	F_(1,60)_ = 12.40 ***	F_(1,60)_ = 43.59 ***	F_(1,60)_ = 224.25 ***
T × t	F_(8,60)_ = 1.69	F_(8,60)_ = 38.74 ***	F_(8,60)_ = 2.13 *	F_(8,60)_ = 1.84	F_(8,60)_ = 2.95 ***	F_(8,60)_ = 1.29
T × a	F_(2,60)_ = 17.11 ***	F_(2,60)_ = 41.26 ***	F_(2,60)_ = 7.05 ***	F_(2,60)_ = 7.01 ***	F_(2,60)_ = 14.59 ***	F_(2,60)_ = 0.14
t × a	F_(4,60)_ = 5.83 ***	F_(4,60)_ = 181.09 ***	F_(4,60)_ = 7.99 ***	F_(4,60)_ = 1.16	F_(4,60)_ = 2.21	F_(4,60)_ = 5.70 ***
T × t × a	F_(8,60)_ = 1.75	F_(8,60)_ = 38.74 ***	F_(8,60)_ = 2.13 *	F_(8,60)_ = 1.84	F_(8,60)_ = 2.95***	F_(8,60)_ = 1.29

* *p* < 0.05, ** *p* < 0.01, *** *p* < 0.001.

## Data Availability

The data presented in this study are available in the article. Additional data are available on request from the corresponding author.
